# Diagnosis and Outcome of Cardiac Paragangliomas: A Retrospective Observational Cohort Study in China

**DOI:** 10.3389/fcvm.2021.780382

**Published:** 2022-01-05

**Authors:** Xueqi Dong, Xu Meng, Ting Zhang, Lin Zhao, Fang Liu, Xu Han, Yecheng Liu, Huadong Zhu, Xianliang Zhou, Qi Miao, Shuyang Zhang

**Affiliations:** ^1^Department of Cardiology, Fuwai Hospital, National Center for Cardiovascular Disease, Chinese Academy of Medical Sciences and Peking Union Medical College, Beijing, China; ^2^Department of Emergency, State Key Laboratory of Complex Severe and Rare Diseases, Peking Union Medical College Hospital, Chinese Academy of Medical Science and Peking Union Medical College, Beijing, China; ^3^Department of Emergency, Puren Hospital of Beijing, Beijing, China; ^4^Department of Cardiac Surgery, State Key Laboratory of Complex Severe and Rare Diseases, Peking Union Medical College Hospital, Chinese Academy of Medical Science and Peking Union Medical College, Beijing, China; ^5^Department of Cardiology, State Key Laboratory of Complex Severe and Rare Diseases, Peking Union Medical College Hospital, Chinese Academy of Medical Science and Peking Union Medical College, Beijing, China

**Keywords:** cardiac paraganglioma, clinical feature, diagnosis, outcome, CPGL

## Abstract

**Background:** Cardiac paragangliomas (CPGLs) are rare neuroendocrine tumors that are easily overlooked and difficult to diagnose. Detailed comprehensive data regarding CPGL diagnosis and outcome are lacking.

**Methods:** We retrospectively analyzed a cohort of 27 CPGL patients. This cohort represents the largest such cohort reported to date.

**Results:** The prevalence of trilogy symptoms (concurrent palpitations, hyperhidrosis, and headache) was frequent (9/27, 33.3%). Sensitivity of echocardiography and contrast-enhanced computed tomography for localization of CPGL were 81.8% and 87%, respectively. Octreotide scintigraphy showed 100% sensitivity for detecting GPCLs, while sensitivity of I^131^-metaiodoben-zylguanidine scintigraphy was only 32.9%. Multiple tumors were found in 29.6% of patients. Most CPGLs originated from the epicardium or root of the great vessels (92.9%) and were mostly supplied by the coronary arteries and their branches (95.7%). Twenty-four patients underwent surgical treatment. Although local invasion was present in 40.0% of patients, it did not affect long-term outcome. Mean follow-up was 6.9 ± 3.6 years. Biochemical remission was achieved in 85% of patients. The recurrence rate was 15%.

**Conclusions:** Manifestations of CPGLs are non-specific and they can be difficult to detect on imaging examinations. Octreotide scintigraphy should be performed in patients with suspected paragangliomas to screen for multiple lesions. Surgical resection of CPGLs can achieve symptom relief and biochemical remission.

## Introduction

Paragangliomas (PGLs) are rare neuroendocrine tumors derived from extra-adrenal autonomic paraganglia (1). Cardiac paragangliomas (CPGLs) originate from neuroectodermally derived paraganglionic cells of the autonomic ganglia of the heart. They account for <0.004% of cases of hypertension and <1% of primary cardiac tumors (2). CPGLs commonly arise from sympathetic paraganglia and tend to be functional, releasing one or more types of catecholamine (epinephrine, norepinephrine, or dopamine). Catecholamine oversecretion can cause cardiovascular and metabolic abnormalities such as elevation of heart rate and blood pressure (BP) and increased cardiac conduction velocity and myocardial contractility. CPGLs may also induce myocardial infarction and dyspnea when surrounding structures are involved, even in patients with nonfunctional tumors (3–5). Most previous CPGL research has focused on surgical treatments including resection and transplantation (6, 7). Although surgical treatment is widely considered the most effective treatment for functional CPGLs, the reported 5-year overall survival is only 78.8% for patients with benign lesions (6) and even lower for those with malignant lesions. We consider delayed diagnosis, which results from the insidious nature of the disease, and lack of disease awareness as main explanations of this poor prognosis. Based on our experience, CPGL can be easily missed in clinical practice, especially when it occurs with multiple other PGLs at the same time. The reported mean disease duration before CPGL diagnosis is almost 10 years; therefore, organ damage and complications may develop before diagnosis (7). Even though CPGL is an extremely rare cause of hypertension (2), it still may be underestimated because a considerable number of patients could be missed (8). Early and accurate diagnosis is important to reduce the incidence of disease-related complications and improve prognosis. Moreover, resection is less difficult when the tumor is diagnosed in earlier stages. Detailed comprehensive data regarding CPGL diagnosis and outcome are lacking. Here, we present a retrospective analysis of 27 CPGL patients. To our knowledge, these patients comprise the largest CPGL cohort ever reported.

## Materials and Methods

### Patients

This retrospective observational cohort study examined patients diagnosed with CPGL at Peking Union Medical College Hospital (PUMCH) and Fuwai Hospital from January 1, 2000 to December 31, 2019. It was conducted in accordance with the Declaration of Helsinki and approved by the Ethics Committee of PUMCH. The requirement for informed consent was waived because of the retrospective nature of the study.

### Diagnosis

PGL was diagnosed in accordance with Endocrine Society practice guidelines (9). Most patients received a pathologic diagnosis after tumor excision. Three patients who did not undergo resection were diagnosed based on elevated urine or plasma catecholamines and functional imaging. CPGL was defined as a PGL originating in the heart chambers, myocardium, pericardium, or root of the great vessels. A PGL was defined as functional when urine or plasma catecholamine levels were elevated higher than the upper limit of the normal reference range; those with normal levels were defined as non-functional. Malignant CPGL was defined as CPGL with evidence of metastasis to non-chromaffin tissues according to the 2004 World Health Organization Classification of Tumors (10). Heart failure was defined as New York Heart Association functional class II to IV, based on the 2018 European Society of Hypertension guidelines (11). Hypertension was defined as systolic BP ≥140 mm Hg and/or diastolic BP ≥90 mm Hg on at least 3 occasions on different days (including at least one out-of-office BP measurement) in the absence of antihypertensive drug administration. Peak blood pressure measurements were identified from hospital medical records.

For measurement of catecholamines, 24-h urine samples were collected and evaluated using high performance liquid chromatography-mass spectrometry (the testing kit was developed by the PUMCH clinical laboratory). Patients with severe hypertension were commenced on antihypertensive agents that did not interfere with catecholamine measurements. A positive catecholamine test was defined as epinephrine >6.42 μg/24 h, norepinephrine >40.65 μg/24 h, or dopamine >330.59 μg/24 h.

^131^I-metaiodoben-zylguanidine (MIBG) scintigraphy was performed as follows: ^131^I-MIBG was injected intravenously at least 30 min after blocking the thyroid gland with 300 mg oral sodium perchlorate. Whole-body scintigraphy, including anterior-posterior and lateral views, and single-photon emission computed tomography (SPECT) of the primary tumor region were performed 24 and 48 h after injection. Planar scintigraphy was acquired using a dual-head whole-body γ-camera (Hawkeye; GE Healthcare, Milwaukee, WI, USA) with a scanning velocity of 20 cm/min.

^18^F-flourodeoxyglucose positron emission tomography (PET)/computed tomography (CT) was performed as follows: Patients received an intravenous injection of ^18^F-flourodeoxyglucose (3.7–7.4 MBq/kg). PET/CT studies were performed using a Biograph™ 64 PET/CT scanner (Siemens, Munich, Germany). Whole-body PET acquisition was started 60 to 90 min after the injection (field of view was from the base of the skull to the middle of the thigh; 2-min emission scan per bed position). Data were reconstructed in an overlapping manner at a 2 mm slice thickness with 1 mm reconstruction increments.

^111^In-diethylenetriaminepentaacetic acid-octreotide scintigraphy (OCT) was performed as follows: ^111^In-pentetreotide was prepared by mixing the two components followed by 30 min of incubation according to manufacturer instructions. The tracer solution (10 μg pentetreotide and ^111^In in the form of InCl_3_) was injected intravenously to deliver a dose of 180–200 MBq. Patients received 1 L of intravenous fluid around the time of injection to ensure adequate hydration. Two planar whole-body scintigrams after 4 and 24 h were acquired; All examinations were performed using a Hawkeye SPECT/CT scanner (GE Healthcare).

### Data Collection

Demographic data, medical history, laboratory findings, and imaging results were retrospectively reviewed by two trained researchers. Genetic testing results (next-generation sequencing) were recorded from patients in whom they were available. Potential duplicates with matching birth year, sex, and clinical features were excluded. BP was evaluated using automatic devices. Disease duration was defined as the time between onset of clinical manifestations and diagnosis. Estimated glomerular filtration rate was calculated based on the Chronic Kidney Disease Epidemiology Collaboration equation (12). Body mass index (BMI) was calculated in kg/m^2^.

### Treatment and Follow-Up

All patients except three CPGL patients were treated with surgical excision after adequate preoperative preparation with α-receptor blockers. Beta-receptor blockers and/or calcium channel blockers were added to control blood pressure if needed. As for follow-up, patients were asked to visit the clinic 1 month after discharge and then every 6 months. Clinical symptoms, 24 h urine catecholamine levels (including three types of hormones: epinephrine, norepinephrine and dopamine) and CT scan were performed in these patients to evaluate the status of disease and outcomes. Primary end points included biochemical remission, tumor recurrence, biochemical recurrence, and cardiovascular disease (CVD). Biochemical remission was defined as normalization of biochemical testing after surgery. Tumor recurrence was defined as PGL reappearance after biochemical remission. Biochemical recurrence was defined as reappearance of endocrinological abnormality after biochemical remission. CVD was defined as myocardial infarction, angina, coronary disease, coronary angioplasty, coronary bypass surgery, and stroke (13). Data regarding patient survival and clinical status were obtained either *via* medical records or detailed telephone interviews.

### Data Analysis

Continuous variables are expressed as means with standard deviation. Categorical variables are expressed as numbers with percentage. The Levene test was used to verify homogeneity of variance for continuous variables across samples. Group differences were compared using the chi-square test or Fisher's exact test for categorical variables and the independent Student's *t*-test for continuous variables. Two-sided *P* < 0.05 was considered significant. Statistical analyses were performed using SPSS software version 23.0 (IBM Corp., Armonk, NY, USA).

## Results

### Patient Characteristics

The present study comprised 27 patients diagnosed with CPGL (14 men and 13 women). Patient characteristics are summarized in Table 1. Mean age at diagnosis was 35.74 ± 13.14 years. Mean duration of symptoms prior to diagnosis was 7.88 ± 6.86 years. Prevalence of hypertension was 88.9%. Three types of hypertension were observed: persistent hypertension, paroxysmal hypertension, and persistent hypertension with paroxysmal exacerbation (Table 1). Persistent hypertension was defined as blood pressure ≥140/90 mm Hg for at least 1 month. Paroxysmal hypertension was defined as normal basal blood pressure with sudden elevations (BP ≥140/90 mm Hg) lasting minutes or hours. Persistent hypertension with paroxysmal exacerbation was defined as persistent hypertension as baseline combined with episodic paroxysms of even higher hypertension lasting minutes or hours.

**Table 1:** Baseline characteristics of the CPGL patients.

**Characteristics**	**Numbers** **(***n*** = 27)**
Age of diagnosis years	35.74 ± 13.14
Gender, male (*n*, %)	14 (51.9%)
Course, months	7.88 ± 6.86
**Vital signs**	
Mean SBP, mm Hg	150.35 ± 25.12
Mean DBP, mm Hg	96.00 ± 19.94
Heart Rate, bpm	92.12 ± 12.15
BMI, Kg/m^2^	22.05 ± 2.03
Drinking	1 (3.7%)
Smoking	1 (3.7%)
**Blood pressure**	
Normal-tension	3 (11.1%)
Persistent hypertension	9 (33.3%)
Paroxysmal hypertension	15 (55.6%)
Persistent hypertension with paroxysmal exacerbation	5 (18.5%)
Mean peak SBP, mm Hg	199.40 ± 38.28
Mean peak DBP, mm Hg	137.92 ± 34.78
**Chief complaint**	
Trilogy	9 (33.3%)
Palpitation	21 (77.8%)
Hyperhidrosis	17 (63.0%)
Headache	15 (55.6%)
Dizziness	7 (25.9%)
Chest pain	4 (14.8%)
Pallor	5 (18.5%)
Gastrointestinal symptom	4 (14.8%)
Incidentalomas	1 (3.7)
Paroxysmal symptoms	26 (96.3%)
Triggers for episodes	11/26 (42.3%)
Catecholamine crisis	8 (32.1%)
Hypertensive crisis	6 (22.1%)
Cardiac shock	1 (3.7)
Torsade de pointes	1 (3.7)
**Family history**	
Familial PPGL	4 (14.8%)
Hypertension	8 (29.6%)

*Bpm, beats per minute; CPGL, cardiac paraganglioma; BMI, body mass index; SBP, systolic blood pressure; DBP, diastolic blood pressure; PHEN, pheochromocytoma; PPGL, phaeochromocytoma and paraganglioma*.

Among the CPGL patients, trilogy symptoms (concurrent palpitations, hyperhidrosis, and headache) was the most common complaint; palpitations and paroxysmal symptoms were reported by 77.8% and 96.3% of patients, respectively. However, most paroxysmal symptoms were not associated with specific triggers. Symptoms mimicking myocardial infarction, such as chest pain concurrent with sweating, diarrhea, and vomiting, occurred in four patients (14.8%). Chronic heart failure was diagnosed in one patient with no other possible cause of heart failure. Catecholamine crisis occurred in 8 patients (29.6%): hypertensive crisis in 6, cardiac shock in 1, and torsade de pointes in 1. Family history of PGL (familial PGL) was present in 4 patients.

### Biochemical and Genetic Testing and Imaging Examinations

Biochemical testing results are shown in Table 2. Twenty-six patients had functional tumors (96.3%). Among these, 88.5% were noradrenergic, 50% were adrenergic, and 42.3% were dopaminergic. The diagnostic value of different imaging examinations is shown in Table 2. Echocardiography and contrast-enhanced computed tomography (CT) showed sensitivities of 81.8 and 87%, respectively, for localization of CPGL (100%). OCT, PET/CT, and magnetic resonance imaging showed 100% sensitivity for CPGL. However, the sensitivity of I^131^-MIBG scintigraphy was only 32.9%.

**Table 2:** Biochemical and imaging examinations of CPGL patients.

**Characteristics**	**Number**	**Mean value**
**Elevated catecholamines**	***n*** **= 26**	
Norepinephrine	23 (88.5%)	76.56 ± 14.32
Epinephrine	13 (50.0%)	7.35 ± 3.61
Dopamine	11 (42.3%)	423.57 ± 43.65
Non-functional PPGL	0 (0%)	
**Sensitivity of imaging examinations**		
Ultrasonography	18/22 (81.8%)	
Contrast-enhanced CT	20/23 (87.0%)	
MRI	16/16 (100%)	
OCT	21/21 (100%)	
I^131^-MIBG	6/14 (32.9%)	
PET-CT	9/9 (100%)	
**Multiple PPGL**	***n*** **= 27**	
Total	8 (29.6%)	
Bilateral PHEN	1 (3.7%)	
Head and neck PGL	6 (22.2%)	
Periaortic PGL	1 (3.7%)	
Bladder PGL	1 (3.7%)	

*Values are the mean ± SD or number (%)*.

*CPGL, cardiac paragangliomas; MRI, cardiac magnetic resonance; hsCRP, high sensitivity C reactive protein; CT, computed tomography; eGFR, estimated glomerular filtration rate; ESR, erythrocyte sedimentation rate; LV, left ventricular; MIBG, metaiodoben-zylguanidine scintigraphy; OCT, octreotide scintiscan; PET, positron emission tomography; PGL, paraganglioma*.

Based on the imaging examinations, eight CPGL patients (29.6%) had multiple PGLs: six (22.2%) had concurrent head and neck PGLs (4 with a carotid body PGL and 2 with both carotid body and glomus jugular PGLs), one had a jugular foramen PGL, and one had a right carotid body PGL, periaortic PGL, and right adrenal pheochromocytoma (PHEO). Next-generation sequencing results were available in four patients. Two patients harbored SDHB mutations: one had a CPGL with concurrent adrenal PHEO and the other had a familial PGL. Genetic testing was negative in the other two patients.

### CPGL Localization and Feeding Vessels

**Figure 1:**
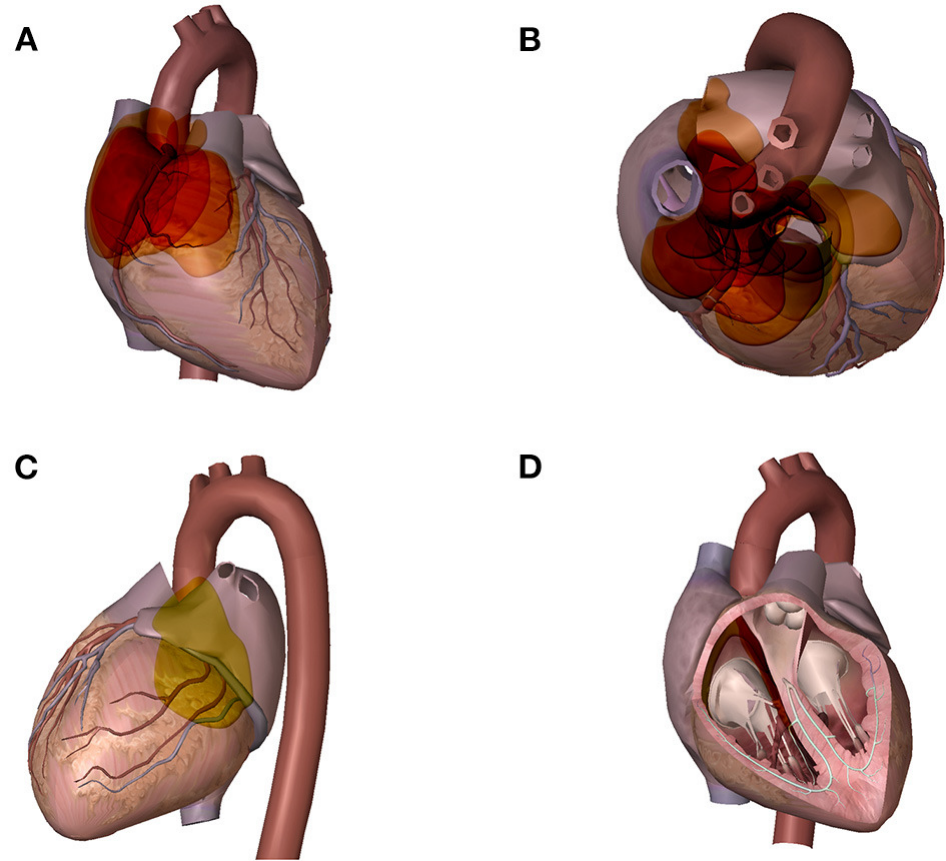
Distribution of GPCLs. A wide variety of the tumor sites could be found in these GPCL patients. Each colored disc represents one tumor, the size, shape, and position of each disc is similar to that of the corresponding tumor. Areas with deeper color indicate areas with higher frequency of tumor. **(A)** cardiac frontal view: tumors located in the interatrial groove and the root of the vena cava **(B)** cardiac axial view: tumors located between the ascending aorta and the main pulmonary artery, and in the root of the vena cava **(C)** cardiac posterior view: tumors located in the atrioventricular groove **(D)** cardiac chamber view: tumors originated from the atrial septum and located in the cardiac chambers.

Twenty-eight tumors were localized among the 27 CPGL group patients (Figure 1). One patient had two lesions: a lateral right atrium lesion and a lesion between the left atrium and right pulmonary artery. Twenty-six CPGL tumors (92.9%) originated from the epicardium or root of the great vessels; the other two originated from the atrial septum and were located in the cardiac chambers. Table 3 shows the localization of CPGLs. The root of the aorta and main pulmonary artery were the most common locations, followed by the root of the vena cava, atrioventricular groove, and interatrial groove. The tumors extended to or compressed adjacent structures in 19 patients (67.9%); these structures included the aortic artery in six (21.4%), pulmonary artery in four (14.3%), vena cava in three (10.7%), right ventricular outflow tract in three (10.7%), atrium in two (7.1%), and principal bronchus in one (3.6%).

**Table 3:** Anatomic characteristics of CPGLs.

**Characteristics**	**Numbers**
Localization	*n =* 28
Intracardial	2 (7.1%)
Interatrial septum	2 (7.1%)
Intrapericardial	26 (92.9%)
Root of Ao	17 (60.7%)
Root of MPA	15 (53.6%)
Superior vena cava	4 (14.3%)
Inferior vena cava	1 (3.6%)
Right atrioventricular groove	3 (10.7%)
Left atrioventricular groove	1 (3.6%)
Interatrial groove	3 (10.7%)
**Coronary angiogram**	
Sensitivity	23/24 (95.8%)
Main feeding vessels	*n =* 23
LCA	2 (8.7%)
LAD	3 (13.0%)
LCX	8 (34.8%)
DIAG	1 (4.3%)
RCA	9 (39.1%)
SN	3 (13.0%)
RIMA	1 (4.3%)

*Ao, aortic; DIAG, diagonal branch; LAD, left anterior descending coronary artery; LCA, left coronary artery, LCX, left circumflex coronary artery; MPA, main pulmonary artery**;** RCA, right coronary artery; RIMA, right internal mammary artery; SN, sinoatrial node artery*.

Coronary angiography was performed in 24 patients and showed 95.8% sensitivity in demonstrating CPGLs. The tumors were fed by one or more arteries but frequently had one major feeding artery (Table 3). Approximately 61% of tumors were supplied by the left coronary artery and its branches (14/23); 52% (12/23) were supplied by the right coronary artery and its branches and 4.3% were supplied by the right internal mammary artery (1/23).

### Treatment and Pathologic Features

Twenty-five of the 28 tumors (89.3%) were surgically excised. Because the CPGL tumors were firmly adherent to adjacent tissues, resection of the involved cardiac tissue and/or vessels followed by complex reconstruction was required. Coronary artery bypass grafting was conducted in 10 patients. The surgical procedures, complications, and intraoperative findings of 17 patients in our cohort have been previously reported in detail (7). Concurrent PGLs were resected in patients with multiple PPGLs. Most patients needed more than one type of antihypertensive agent (mean number, 1.87) for adequate blood pressure control.

Table 4 shows the preoperative medical preparation and pathologic characteristics of the study patients. Maximal CPGL diameter ranged from 35 to 100 mm; tumor weight ranged from 4.6 to 105 g. CPGLs were usually well-defined (98.9%) with complete or partial capsules. Local invasion was common (10 patients, 40.0%); however, malignant CPGL was not diagnosed. CPGL specimens showed the typical microscopic PGL structure and were composed mainly of chief cells assembled in cell clusters or in an organoid fashion with surrounding capillary networks. Pigmentation was observed in one CPGL, which may have indicated recurrent bleeding within the tumor.

**Table 4:** Treatment and follow-up of the CPGL patients.

**Characteristics**	**CPGL patients**
**Number of antihypertensive agents**	1.87 ± 0.69
**Preoperative medical preparation**	*n =* 27
α	7 (25.7%)
α + β	12 (44.4%)
α + CCB	0 (0%)
α + β + CCB	4 (14.8%)
**Pathology**	*n =* 25
Weight, *g*	45.85 ± 28.28
Diameter, *mm*	61.70 ± 16.32
Complete capsule	18 (72.0%)
Local invasion	10 (40.0%)
Well defined	24 (96.0%)
**Pathological diagnosis**	*n =* 27
Benign PPGL	24 (96.0%)
Malignant PPGL	1 (4.3%)
**Follow-up**	*n =* 20
Follow-up duration, years	6.9 ± 3.6
Hypertension	4 (20.0%)
Biochemical remission	17 (85.0%)
Biochemical recurrence	3/17 (17.6%)
Recurrence of tumor	2/17 (11.8%)
New-onset of CVDs	0 (0%)
Death	0 (0%)

*Values are the mean ± SD or number (%)*.

*PPGL, phaeochromocytoma and paragangliomas*.

### Follow-Up

One patient died from bleeding complications and cardiac shock 2 days after surgery. Excluding this patient, follow-up data were available in 20 patients (76.9%). Mean follow-up was 6.9 ± 3.6 years. All patients with follow-up data underwent surgical excision. Biochemical remission was achieved in 85% patients as evaluated by 24-h urinate catecholamine levels; all patients who achieved biochemical remission were also without paroxysmal symptoms. Three patients were diagnosed with recurrence, which occurred at 2, 12, and 14 years of follow-up, respectively. No new-onset CVD nor death occurred during the follow-up period. Table 5 shows the patient follow-up data according to presence of local invasion.

**Table 5:** Follow-up of the subjects with and without local-invasion of CPGL patients.

**Follow-up data**	**With local-invasion**	**Without local-invasion**	***P*** **-value**
	**(***n =*** 8)**	**(***n =*** 12)**	
Hypertension, *n* (%)	1 (12.5%)	3 (25.0%)	0.909
Biochemical remission, *n* (%)	7(87.5%)	10 (80.0%)	0.701
Biochemical recurrence, *n* (%)	2/7 (28.6%)	1/10 (10.0%)	0.732
Recurrence of tumor, *n* (%)	2/7 (28.6%)	0/10 (0%)	0.301
New-onset of CVDs, *n* (%)	0 (0%)	0 (0%)	–
Death, *n* (%)	0 (0%)	0 (0%)	–

## Discussion

To our knowledge, this study represents the largest reported cohort of CPGL patients to date. The main findings were as follows: (1) Hypertension is the main manifestation of CPGL; approximately half of the CPGL patients in our study exhibited paroxysmal hypertension. (2) Most CPGLs are functional and predominately noradrenergic. (3) OCT, PET/CT and magnetic resonance imaging are highly sensitive for detection of CPGLs. (4) Most CPGLs originate from the epicardium or root of the great vessels and are mainly supplied by coronary arteries and their branches. (5) Surgical resection of CPGLs can achieve symptom relief and biochemical remission. Our findings are in line with the those of Wang et al. (6), who reviewed the clinical characteristics of 158 GPCL cases retrieved from the PubMed database. According to their report, most CPGLs are functional and 77.3% of GPCL patients manifest catecholamine-related symptoms. In addition, they noted that tumors were often within the pericardium and mostly arose from the epicardium and root of the great vessels. Furthermore, surgery was the most effective therapeutic strategy.

Compared with previous studies in PHEO patients, the CPGL patients in our study were diagnosed at a younger age and had a longer disease course prior to diagnosis. We hypothesize that CPGL patients are diagnosed younger because CPGL clinical manifestations are more severe; however, because the diagnosis of CPGL is more difficult, the disease duration before diagnosis is longer. We also found that the prevalence of trilogy symptoms was higher in CPGL patients than in PHEO patients, and a higher proportion of CPGL patients experienced palpitations. Previous studies have found that one-fourth of PHEOs are discovered incidentally during imaging examinations for unrelated disorders, while more than three-fourths of CPGL patients exhibit catecholamine-related symptoms (6, 14). Continuing heart beats can be strong stimulations which can induce more frequent secretion of catecholamines than PHEOs. This may explain why GPCL patients manifest more severe clinical symptoms, face higher incidence of catecholamine crisis, and with a higher level of blood pressure than PHEO patients.

Also, we considered that CPGL patients without obvious symptoms may be missed because CPGL is not considered in the differential diagnosis or the CPGL is not detected on common imaging examinations. CPGL-induced chest pain can mimic myocardial infarction chest pain (15). In our cohort, four CPGL patients exhibited angina-like chest pain combined with palpitations, dyspnea, and/or pallor. Chest pain can be caused by multiple factors. Tumor compression of the coronary artery, catecholamine-stimulated palpitations, and coronary artery spasm may cause decreased myocardial blood supply. In addition, cardiac tumor-induced blood flow shunting may induce or exacerbate myocardial ischemia. Moreover, concurrent coronary artery disease may mask CPGL manifestations. Therefore, trilogy symptoms as well as angina-like chest pain may suggest the presence of a CPGL.

CPGLs are diagnosed with the same diagnostic procedures used for other types of PGLs (9). Qualitative diagnoses depend on levels of catecholamines or their metabolites in urine, followed by imaging examinations to determine location. In our cohort, noradrenergic tumors were most frequent, followed by adrenergic and dopaminergic tumors. All CPGLs were functional except for one found incidentally. Fewer CPGLs are found incidentally because they are more difficult to detect using routine methods such as ultrasonography and CT. In our study, echocardiography and contrast-enhanced CT were most frequently used for tumor detection. However, both showed unsatisfactory sensitivity for CPGL detection. In a previous review, the sensitivity of CT for CPGL detection was ~80% and ultrasonography and CT exhibited poor ability to detect tumors in unusual locations, such as the carotid body and glomus jugulare (6). Therefore, functional imaging plays an important role in identifying tumor location. OCT and I^131^-MIBG are common imaging modalities used to detect neuroendocrine neoplasms. MIBG is a radiopharmaceutical agent that accumulates in catecholamine-producing cells. MIBG labeled with I^123^ or I^131^ is widely used to detect PGLs. Its sensitivity ranges from 85 to 88 % for PHEOs and from 56 to 75% for PGLs; specificity ranges from 70 to 100% for PHEOs and from 84 to 100% for PGLs (16–19). In our study, ^131^I-MIBG showed only a 32.9% sensitivity for detecting CPGL; in contrast, OCT showed a higher sensitivity (100%). The sensitivity of MIBG for CPGL detection was 75% in a previous review that examined 158 sporadic cases, which was considerably higher than our result; however, their OCT sensitivity result was the same as ours. Furthermore, nearly one-third of the CPGL patients in our study had multiple PGLs. Therefore, we suggest that OCT is necessary for patients with suspected PGLs, even when ultrasonography and CT are negative. The current gold-standard in functional imaging for PGLs is18F-FDOPA PET/CT, our study also showed 100% sensitivity of PET-CT. According to a study by Ingo Janssen et al., 68Ga-DOTATATE PET/CT identified more lesions and showed a higher sensitivity in the localization of PGLs than 18F-FDOPA PET/CT (20). However, high costs of PET/CT limits its use in clinical practice.

Because CPGLs are mainly supplied by the coronary arteries, preoperative coronary angiography is useful. In our study, coronary angiography showed 95.8% sensitivity for detection, 95.7% of tumors had a coronary artery blood supply, and nearly half of the patients underwent coronary artery bypass grafting during tumor resection. These findings are in agreement with those of a previous study (6). CPGLs originate mostly along the distribution of the cardiac plexus (6). In our cohort, most originated from the epicardium and root of the great vessels; however, two arose in the atrial septum and were located within the cardiac chambers, which is extremely rare (21). Compression of adjacent structures is common in CPGLs and may lead to symptoms that mimic myocardial infarction (coronary ostia compression), heart failure (valve obstruction), syncope (valve obstruction and great vessel compression), and chest distress (principal bronchus compression), which can make accurate diagnosis difficult. CPGL should be suspected in patients with such manifestations and trilogy symptoms or other paroxysmal symptoms.

Surgical treatment is suggested for all PGLs (9), including CPGLs. In our cohort, 85% of patients achieved biochemical remission after surgical treatment, all of whom were alive without symptoms at last follow-up. The recurrence rate was 15%. These findings are in line with previous studies in GPCL patients. In our cohort, 10 tumors exhibited local invasion on postoperative pathologic examination; however, local invasion had no impact on long-term outcome. A previous study also showed that local invasion does not predict malignancy in PGLs (22). According to the 2004 World Health Organization Classification of Tumors, evidence of metastasis to distant non-chromaffin sites is the only criterion for PGL malignancy (10). In patients with malignant PGL, adjunctive chemotherapy and radiotherapy are often used after surgery; however, their effects remain uncertain. Further study is warranted. Table 6 summarizes the clinical features of 9 previously reported malignant CPGLs.

**Table 6:** Cases of malignant cardiac paragangliomas reported in the previous literatures.

**Patient no**.	**Gender**	**Age (years)**	**Tumor location**	**Tumor size (cm)**	**Metastatic lesion**	**Treatment**	**Outcome/follow-up**	**Catecholamine**	**Genetics**
1	F	58	At the inferior vena cava and the right atrium.	NA	Lung	Su	Alive 20 months after diagnosis	NA	NA
2	M	25	At the roof of the atria and penetrating into the right atrium and right ventricle.	8 × 6 × 8	Bone	Ra	NA	Normal	NA
3	F	59	Behind the root of the aorta below the pulmonary trunk.	6	Bone	Ch	Died 4 years after diagnosis	NE	NA
4	M	35	At the roof of the left atrium and attaching to the left main coronary artery.	4 × 2.5 × 2	Lymph nodes	Su, Ch	Alive 5 years after diagnosis	NE	NA
5	M	13	Extending from the right to the left superior pulmonary veins to the inferior border of the pulmonary artery, and caudally extending between the inferior pulmonary veins.	6	Lymph nodes	Su	Alive 2 years from the initial operation	TC	NA
6	M	39	Left atrial cavity.	7	Brain	Su, Ra	Died at 6 months postoperatively	NE, DA, TC	NA
7	M	27	Surrounding the atria cordis.	NA	Bone	Su, Ra	Died at 20 months postoperatively	NA	NA
8	M	59	Right atrial.	NA	Bone, liver, lungs, brain and lymph nodes	Su, Ch	Died 5 years after diagnosis	NE	SDHB (–),break SDHD (–)
9	F	51	Behind the posterior wall of the heart.	NA	Bone	NA	NA	Normal	SDHC (+)

*Ch, Chemotherapy; DA, Dopamine; E, Epinephrine; F, female; M, male; NA, not available; NE, Norepinephrine; Su, Surgical resection; Ra, Radiotherapy; TC, Total catecholamine*.

Genetic testing is recommended for all PGL patients, not only because genetic diagnosis provides important information for clinical management, prognosis, and translational research, but also to increase understanding of the hereditary nature of these tumors. Currently, 22 genes have been linked to the pathogenesis of PGLs, and 30% to 40% of PGLs are hereditary (23–26). Among CPGL patients, mutations in SDHB and SDHD have been most commonly reported; mutations in PGL-associated genes such as the rearranged during transfection and von Hippel-Lindau genes have not been detected (6). Mutations in SDHB are associated with higher risk of aggressive behavior than other SDH subtypes (26–28). Only four patients in our cohort underwent genetic testing because of the high cost; two carried SDHB mutations.

Our study is limited by its low rate of genetic testing, relatively small sample size, and retrospective design, which may have introduced selection bias. However, to our knowledge, our cohort is still the largest one reported to date.

## Conclusions

Most CPGLs are functional and noradrenergic. Nearly one-third of patients with CPGLs also harbor another PGL. The majority of CPGLs originate from the epicardium or root of the great vessels and are mainly supplied by the coronary arteries and their branches. The ability of echocardiography, contrast-enhanced CT, and MIBG to detect CPGLs is poor. However, OCT and PET-CT are highly sensitive. We suggest performing OCT in patients with suspected PGLs to screen for multiple lesions. Surgical resection of CPGLs can achieve symptom relief and biochemical remission.

## Data Availability Statement

The original contributions presented in the study are included in the article/supplementary material, further inquiries can be directed to the corresponding author/s.

## Ethics Statement

The studies involving human participants were reviewed and approved by the Ethics Committee of PUMCH. The requirement for informed consent was waived because of the retrospective nature of the study.

## Author Contributions

XD and XM collected patients' data, performed the analyses, and wrote the paper. TZ assisted with data collection and analysis. LZ, FL, and XH assisted with data collection. YL, HZ, and XZ designed the study. QM and SZ assisted with study design. All authors read and approved the final manuscript.

## Funding

This work was supported by the National Key Research and Development Plan of China (grant to XZ, grant number 2016YFC1300100) and the Non-Profit Central Research Institute Fund of the Chinese Academy of Medical Sciences (grant to YL, grant number 2019XK320035).

## Conflict of Interest

The authors declare that the research was conducted in the absence of any commercial or financial relationships that could be construed as a potential conflict of interest.

## Publisher's Note

All claims expressed in this article are solely those of the authors and do not necessarily represent those of their affiliated organizations, or those of the publisher, the editors and the reviewers. Any product that may be evaluated in this article, or claim that may be made by its manufacturer, is not guaranteed or endorsed by the publisher.

## Abbreviations

CPGLs: cardiac paraganglioma
PHEO: pheochromocytoma
PGL: paraganglioma
PUMCH: Peking Union Medical College Hospital
MIBG: I131-metaiodoben-zylguanidine
SPECT: single-photon emission computed tomography
PET: positron emission tomography
CT: computed tomography
OCT: octreotide scintigraphy
CVD: cardiovascular disease.
